# Supplementary material for: Handgrip strength cutoff value predicting successful extubation in mechanically ventilated patients
Source: PLoS One. 2021 Oct 21;16(10):e0258971. doi: 10.1371/journal.pone.0258971 (PMC8530306; doi:10.1371/journal.pone.0258971)
Supplement: S1 Table — (DOCX) [file pone.0258971.s001.docx]

**S1 Table.** Comparison of baseline characteristics, mechanical ventilation data, rapid shallow breathing index, and handgrip strength between 3 weaning groups

| **Characteristic** | **Simple weaning**  **n=72** | **Difficult weaning**  **n=19** | **Prolonged weaning**  **n=2** | **P-value** |
| --- | --- | --- | --- | --- |
| Age, years | 71.2 ± 16.2 | 72.2 ± 11.8 | 81.0 ± 4.2 | 0.661 |
| Male | 38 (52.8) | 14 (73.7) | 2 (100) | 0.124 |
| Body mass index, kg/m^2^ | 23.4 ± 4.6 | 23.8 ± 4.9 | 22.4 ± 1.4 | 0.908 |
| APACHE II, points | 13.1 ± 4.7 | 15.4 ± 4.5 | 11.2 ± 2.1 | 0.133 |
| Diabetes | 58 (80.6) | 11 (57.9) | 1 (50.0) | 0.089 |
| Pneumonia | 27 (37.5) | 8 (42.1) | 2 (100) | 0.199 |
| Chronic heart failure | 8 (11.1) | 1 (5.3) | 0 (0) | 0.668 |
| COPD | 10 (13.9) | 1(5.3) | 0 (0) | 0.510 |
| Malignancy | 8 (11.1) | 5 (26.3) | 0 (0) | 0.20 |
| MV duration before first weaning trial, days | 16.4 ± 7.2 | 13.7 ± 4.7 | 15.1 ± 0.4 | 0.426 |
| PSV | 56 (77.8) | 13 (68.4) | 0 (0) | 0.313 |
| T-piece | 16 (22.2) | 6 (31.6) | 2 (100) | 0.572 |
| RSBI*, breaths/min/L | 39.3 ± 9.7 | 43.6 ± 6.7 | 39.0 ± 1.4 | 0.202 |
| HSG*, kg | 16.36 ± 7.17 | 13.73 ± 4.69 | 15.1 ± 0.42 | 0.318 |

Data are presented as n (%), mean±SD

APACHE II=acute physiology and chronic health evaluation II, COPD=chronic obstructive pulmonary disease, HGS=handgrip strength, MV=mechanical ventilation, PSV=pressure support ventilation, RSBI=rapid shallow breathing index

*Tests were performed at 10 minutes before spontaneous breathing trial.
