# Supplementary material for: Handgrip strength cutoff value predicting successful extubation in mechanically ventilated patients
Source: PLoS One. 2021 Oct 21;16(10):e0258971. doi: 10.1371/journal.pone.0258971 (PMC8530306; doi:10.1371/journal.pone.0258971)
Supplement: Attachment — Submitted filename: Response to Reviewers_v1.docx [file pone.0258971.s002.docx]

Dear Reviewers of *PLOS ONE*:

I submitted an original contribution to *PLOS ONE* entitled “*Handgrip Strength Cutoff Value Predicting Successful Extubation in Mechanically Ventilated Patients*” (Manuscript Number PONE-D-21-10826). You have kindly expressed some interesting points with your comments. Therefore, my response to the comment is below.

**RESPONSE TO REVIEWER 1:**

**REVIEWER COMMENT 1:** I just wonder why more than 80% of patients were in general medical wards.

**RESPONSE:** I have added more texts to explain the reasons in Paragraph 1 of Discussion section.

**REVIEWER COMMENT 2:** Please briefly describe study sites, including both general ward and ICU.

**RESPONSE:** I have added more descriptions in Paragraph 1 of Methods section (Study design and participants).

**REVIEWER COMMENT 3:** Please add something more in the table 2, including patients’ characteristics, use of sedation, neuromuscular blockage and steroid.

**RESPONSE:** I have added more data including patient characteristics in Table 2.

**REVIEWER COMMENT 4:** We need more discussion about HSG, not only the description of each study’s finding about HSG.

**RESPONSE:** I have added more discussions in Paragraph 1 to 4 of Discussion section.

**RESPONSE TO REVIEWER 2:**

**REVIEWER COMMENT 1:** SBT failure and extubation failure should be considered different outcomes.

**RESPONSE:** I have added data of extubation failure as weaning outcome in Table 1. **REVIEWER COMMENT 2:** The variation in the SBT duration (30 to 120 minutes) can interfere in the outcome, and even in the HGS result. For some patients, 120 min is a big challenge. The standardization of the SBT duration is very important (all patients with 30 min or all with 120 min). Another possibility is to analyze separated those with 30 min form those with 120 min.

**RESPONSE:** I have added more data on SBT duration in Table 1 and Table 2.

**REVIEWER COMMENT 3:** The type of SBT performed can affect the results. This method should be standardized or analyze separated patients test in PSV and those tested in t-piece.

**RESPONSE:** I have added PSV data in Table 2.

**REVIEWER COMMENT 4:** How many measures of the HGS was performed? Do you consider the first try? There was a training time or explanation?

**RESPONSE:** I have added more details in Methods section (Measurements).

**REVIEWER COMMENT 5:** How did you define the patient’s capacity to perform the HGS? Glasgow coma scale? RASS? The capacity to follow 2 o 3 commands?

**RESPONSE:** I have added more details in Paragraph 1 of Methods section (Study design and participants).

**REVIEWER COMMENT 6:** How can you explain/discuss the low ROC value of RSBI in this cohort? This is very different from others studies that used RSBI.

**RESPONSE:** I have added more discussions in Paragraph 2 of Discussion section.

I appreciate your consideration of my manuscript for publication in *PLOS ONE*.

Sincerely,

Narongkorn Saiphoklang, M.D.
