# Supplementary material for: Handgrip strength cutoff value predicting successful extubation in mechanically ventilated patients
Source: PLoS One. 2021 Oct 21;16(10):e0258971. doi: 10.1371/journal.pone.0258971 (PMC8530306; doi:10.1371/journal.pone.0258971)
Supplement: Attachment — Submitted filename: Response to Reviewers.docx [file pone.0258971.s003.docx]

Dear Editors of *PLOS ONE*:

I submitted an original contribution to *PLOS ONE* entitled “*Handgrip Strength Cutoff Value Predicting Successful Extubation in Mechanically Ventilated Patients*” (Manuscript Number PONE-D-21-10826). Thank you for your review and comments regarding my manuscript. I would like to explain and submit a revised manuscript. My response to the comment is below.

**RESPONSE TO REVIEWER 1:**

**REVIEWER COMMENT:** The authors response well, so I have no more comment. Therefore, I recommend the present manuscript can be accepted.

**RESPONSE:** I would like to gratefully thank the reviewer for kindly reviews.

**RESPONSE TO REVIEWER 2:**

**REVIEWER COMMENT 1:** The outcome used in table 2 is weaning success and failure, but the outcome described in methods and even mentioned in the title is extubation success or failure. Weaning success and extubation success are two different outcomes. Weaning success is defined as to pass for the SBT without any sign of intolerance, while extubation success is to breathe spontaneously for 48 hours without NIV dependence or reintubation. Authors must standardize the outcome used.

**RESPONSE:** I have added the definition of extubation failure for this study in Method section, paragraph 5. Also, I have changed the weaning outcome to new words; extubation success and extubation failure, in Table 1, the heading title of Table 2, and Figure 1.

**REVIEWER COMMENT 2:** Regarding my previous comment “The type of SBT performed can affect the results. This method should be standardized or analyze separated patients test in PSV and those tested in t-piece.”

There was difference in the outcome in patients who performed the SBT in PSV or T piece. If you analyze the HGS in this 2 groups separately, the result would be the same?

**RESPONSE:** I have added T-piece data as the weaning mode in Table 2 which is separated from PSV data.

**REVIEWER COMMENT 3:** Results section – first paragraph – “weaning success was 6.5%” – I suppose you mean “weaning failure”.

**RESPONSE:** I have corrected this word to “extubation failure” in Results section. Also, I have corrected to new words in Abstract section, Results section, the heading title of Table 2 and 3, and Figure 1.

I appreciate your consideration of my manuscript for publication in *PLOS ONE*.

Sincerely,

Narongkorn Saiphoklang, M.D.
