# Supplementary material for: Handgrip strength cutoff value predicting successful extubation in mechanically ventilated patients
Source: PLoS One. 2021 Oct 21;16(10):e0258971. doi: 10.1371/journal.pone.0258971 (PMC8530306; doi:10.1371/journal.pone.0258971)
Supplement: Attachment — Submitted filename: Response to Reviwers.docx [file pone.0258971.s004.docx]

**RESPONSE TO EDITOR**

**EDITOR COMMENT:** *Overall, the manuscript is improved in its current version. I would recommend adding a brief acknowledgement in the limitations section about the relatively small sample size--while the point estimates indicate good accuracy, the CIs are wide and thus the results should be cautiously interpreted.*

**RESPONSE: Thank you for your kindly comments. I have added this sentence in the limitations section.**

I appreciate your consideration of my manuscript for publication in *PLOS ONE*.

Sincerely,

Narongkorn Saiphoklang, M.D.
